# Brain state and polarity dependent modulation of brain networks by transcranial direct current stimulation

**DOI:** 10.1002/hbm.24420

**Published:** 2018-10-30

**Authors:** Lucia M. Li, Ines R. Violante, Rob Leech, Ewan Ross, Adam Hampshire, Alexander Opitz, John C. Rothwell, David W. Carmichael, David J. Sharp

**Affiliations:** ^1^ Computational, Cognitive, and Clinical Imaging Lab, Division of Brain Sciences, Department of Medicine Imperial College London UK; ^2^ Sobell Department of Motor Neuroscience and Movement Disorders UCL Institute of Neurology, University College London London UK; ^3^ School of Psychology University of Surrey Guildford UK; ^4^ Centre for Neuroimaging Science Kings College London UK; ^5^ Department of Biomedical Engineering University of Minnesota Minneapolis Minnesota; ^6^ Department of Biomedical Engineering Kings College London UK

**Keywords:** default mode network, magnetic resonance imaging, salience network, stimulation

## Abstract

Despite its widespread use in cognitive studies, there is still limited understanding of whether and how transcranial direct current stimulation (tDCS) modulates brain network function. To clarify its physiological effects, we assessed brain network function using functional magnetic resonance imaging (fMRI) simultaneously acquired during tDCS stimulation. Cognitive state was manipulated by having subjects perform a Choice Reaction Task or being at “rest.” A novel factorial design was used to assess the effects of brain state and polarity. Anodal and cathodal tDCS were applied to the right inferior frontal gyrus (rIFG), a region involved in controlling activity large‐scale intrinsic connectivity networks during switches of cognitive state. tDCS produced widespread modulation of brain activity in a polarity and brain state dependent manner. In the absence of task, the main effect of tDCS was to accentuate default mode network (DMN) activation and salience network (SN) deactivation. In contrast, during task performance, tDCS increased SN activation. In the absence of task, the main effect of anodal tDCS was more pronounced, whereas cathodal tDCS had a greater effect during task performance. Cathodal tDCS also accentuated the within‐DMN connectivity associated with task performance. There were minimal main effects of stimulation on network connectivity. These results demonstrate that rIFG tDCS can modulate the activity and functional connectivity of large‐scale brain networks involved in cognitive function, in a brain state and polarity dependent manner. This study provides an important insight into mechanisms by which tDCS may modulate cognitive function, and also has implications for the design of future stimulation studies.

## 1 INTRODUCTION

Transcranial direct current stimulation (tDCS) has been extensively used in an attempt to modulate cognitive function in both healthy and disease populations (Coffman, Clark, & Parasuraman, 2014). However, the behavioral results are variable and a recent meta‐analysis concluded that single‐session tDCS produces no effect on a range of cognitive tasks (Horvath, Forte, Carter, & Horvath, 2015). This has fuelled skepticism about whether tDCS has any potential to modulate brain activity and cognitive function.

Understanding the behavioral effects of tDCS has been limited by a lack of mechanistic understanding of how it affects the brain, particularly at the level of the large‐scale brain networks whose coordinated action mediate cognitive function (Bressler & Menon, 2010; Mesulam, 1990). For example, it is unclear whether stimulation interacts with underlying brain activity and whether short duration stimulation is sufficient to influence brain activity. The effect of stimulation polarity on cognitive brain networks is a particularly important aspect of tDCS which is currently poorly understood. Studies of the primary motor cortex (M1) suggest that anodal and cathodal tDCS have opposite effects on neuronal excitability (Nitsche et al., 2005). However, previous cognitive studies have not examined the effect of polarity on network activation and motor effects of stimulation cannot necessarily be extrapolated to cognitive brain networks. Moreover, the influence of polarity on cognitive network function and behavior is poorly characterized, and there have been no studies which have investigated how polarity interacts with an underlying cognitive brain state. Other factors, such as brain state, may also influence the effects of stimulation (Lefebvre & Liew, 2017; Li, Uehara, & Hanakawa, 2015). It is therefore important to directly investigate the effects of tDCS on large‐scale brain network activity to clarify the mechanism by which tDCS may influence behavior.

Concurrent tDCS/functional MRI (fMRI) is an ideal method for studying the physiological effects of stimulation but, to date, such studies are few in number. Previous tDCS/fMRI studies have focused on the motor system. These have shown that that tDCS can modulate brain activity, as measured by hemodynamic changes, with effects seen remote from the site of stimulation measured by Blood Oxygenation Level Dependent (BOLD) fMRI (Amadi, Ilie, Johansen‐Berg, & Stagg, 2014; Antal, Polania, Schmidt‐Samoa, Dechent, & Paulus, 2011; Polania, Paulus, & Nitsche, 2012; Sehm, Kipping, Schäfer, Villringer, & Ragert, 2013; Stagg & Johansen‐berg, 2013). A previous study using high‐density tDCS (HD‐tDCS), which permits multiple cephalic sites to be simultaneously targeted, also suggests that modulation of whole networks is possible and behaviorally relevant (Turski et al., 2017). This study provided some evidence that changes in EEG power were correlated with performance on a cognitive battery.

**Figure 1:**
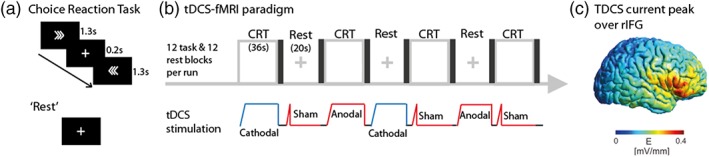
(a) Stimuli in the choice reaction task. (b) The tDCS/fMRI paradigm: each run comprised four blocks each of CRT + anodal, CRT + cathodal, CRT + sham, “rest” + anodal, “rest” + cathodal, and “rest” + sham tDCS. Each block was followed by a brief period of black screen and no stimulation. Each participant had three runs (performing 36 task blocks and 36 rest blocks in total). (c) Modeling showing peak current density over the rIFG [Color figure can be viewed at https://wileyonlinelibrary.com]

Performance of a range of cognitive tasks is associated with activation of the salience network (SN) and concurrent deactivation of the default mode network (DMN; Buckner, Andrews‐Hanna, & Schacter, 2008; Hampshire & Sharp, 2015; Raichle et al., 2001; Seeley et al., 2007). Here we investigate the physiological effects of tDCS on these large‐scale cognitive networks using simultaneous tDCS/fMRI. We employed a factorial design to manipulate both cognitive state (choice reaction task [CRT] or “rest”) and stimulation polarity (anodal or cathodal) (Figure 1). A novel protocol based on short stimulation durations (in seconds) enabled a direct comparison between the effects of different polarities during different cognitive brain states, and interactions between brain state and polarity. The CRT produces robust and stable SN and DMN anti‐correlation (Sharp et al., 2011) and its cognitive simplicity minimizes learning effects. This enabled us to examine the neurophysiological effects of tDCS while diminishing the risks of possible confounds arising from BOLD signal changes related to variability in performance.

We investigated the effects of stimulating the right inferior frontal gyrus (rIFG) (Figure 1c). This region and the underlying anterior insula form part of the SN, which additionally comprises dorsal anterior cingulate cortex and presupplementary motor area (dACC/pre‐SMA). Activity of the rIFG is seen in a range of cognitive contexts and the region is thought to influence activity in other cognitive regions, acting as a switch between different cognitive states (Hampshire & Sharp, 2015; Mesulam, 1990; Sridharan, Levitin, & Menon, 2008), and influencing activity within the more extensive frontoparietal control network (FPCN) as well as anti‐correlated activity within the DMN (Fox et al., 2005). Hence, we tested the hypotheses that: (a) tDCS to the rIFG can modulate the activity and connectivity of intrinsic large‐scale brain networks relevant to cognitive function; (b) the effects of tDCS will interact with cognitive brain state, and that (c) anodal and cathodal tDCS will have distinctive effects on cognitive network activity.

## 2 METHODS

### 2.1 Participants

We recruited healthy volunteers from the Imperial College Clinical Research Facility healthy volunteers list, with no history of neurological or psychiatric illness (*n* = 26, 13F:13M; mean age = 38 years, *SD* = 15.5 years). All volunteers gave written informed consent. The study conforms to the Declaration of Helsinki and ethical approval was granted through the local ethics board (NRES Committee London, West London & GTAC). All participants were naïve to tDCS.

### 2.2 tDCS–fMRI task and paradigm

Functional MRI was acquired while participants performed a blocked Choice Reaction Task (Figure 1). This is a forced choice task where participants press a button with the right or left index finger, to the presentation of a left or right pointing arrow. The task was programmed in MATLAB (MathWorks, Natick, MA) using Psychtoolbox (Brainard, 1997) and responses were recorded through a fiber‐optic response box (NordicNeuroLab, Bergen, Norway), interfaced with the stimulus presentation PC. A trial consisted of a central fixation cross presented for 200 ms, followed by an arrow presented centrally for 1,300 ms during which the participant was required to respond. Each run had 12 task blocks (each block lasted 36 s, and comprised 24 trials) and 12 rest blocks (fixation cross, 20 s), interspersed with brief periods of black screen (4.87 or 3.11 s) during which participants had been instructed to look straight ahead. The black screen durations were different to introduce jitter into the paradigm. During each block, participants received anodal, cathodal, or sham tDCS, resulting in a factorial design, consisting of four blocks of six possible conditions: “rest” + sham; “rest” + anodal; “rest” + cathodal; CRT + sham; CRT + anodal; CRT + cathodal. The order of the blocks was pseudorandomized but the same across all participants. Each participant performed three runs sequentially, with a brief 2–3 min rest between acquisitions to prevent fatigue. Across the three runs, participants received a total of 18 min of full intensity tDCS.

Participants also performed a separate shorter blocked CRT, with no tDCS, prior to the tDCS–fMRI paradigm to determine the basic patterns of BOLD activity during task performance. The CRT was selected because we were specifically investigating the effect of stimulation on cognitive brain networks, and we have previously demonstrated that the CRT produces activation of the SN and deactivation of the DMN, which are robust and stable across time (Sharp et al., 2011).

### 2.3 Statistical analysis of behavioral results

Statistical analyses of task performance were conducted using MATLAB (MathWorks, Natick, MA) and R (http://www.r-project.org). We calculated accuracy (defined as the percentage of correct responses and modeled individual overall reaction times (RT) and first RTs (the RT of the first trial within each block) with an exGaussian distribution (Lacouture & Cousineau, 2008).

### 2.4 Delivery of tDCS

Stimulation was delivered using an MR‐compatible battery‐driven stimulator (NeuroConn GmbH, Ilmenau, Germany) with a previously described circuit (Violante et al., 2017). The “active” electrode (4.5 cm diameter circular rubber electrode) was placed over F8 (based on the 10–20 EEG International system), which corresponds to the pars triangularis of the rIFG, and the “return” electrode (7 × 5 cm rectangular rubber electrode) was placed on the right shoulder with the longitudinal axis parallel to the coronal plane (center of electrode placed over midpoint between tip of the acromion and base of neck). An extracephalic position for the return electrode was selected in order to avoid delivering a current of opposite polarity to another cortical region, as this may have caused additional network effects that would have been difficult to separate from stimulation to the rIFG. Although there are theoretical concerns that extracephalic references may be potentially unsafe in participants with cardiovascular conditions due to the effects of current passing through the brainstem, this effect on autonomic balance is by no means certain. Indeed, it has been previously found that an extracephalic reference montage did not induce any such changes in healthy participants (Vandermeeren, Jamart, & Ossemann, 2010). In our study, a medically qualified researcher screened all participants and excluded anyone with cardiovascular medical histories.

Anodal and cathodal tDCS was delivered with a ramp of 4.5 s up to 1.8 mA, full intensity stimulation and a ramp down over 0.5 s. Sham tDCS consisted of the ramp up and down stages only. About 1.8 mA (rather than 2 mA) was used to maintain safe impedance readings during simultaneous tDCS–fMRI using this equipment. Electrodes had a layer of conductive paste (Ten20, D.O. Weaver, Aurora, CO), which held them in place and reduced impedances. Prestimulation impedances were below 3 kΩ and maximum impedance during stimulation was 29 kΩ. The stimulator was controlled by a National Instruments DAQ device (National Instruments, Newbury, UK), receiving output from in‐house MATLAB scripts. Heart rate was monitored concurrently in 23 participants using the pulse oximetry of the integrated Siemens Physiological Monitoring Unit. The setup and subsequent signal analysis have been previously described (Violante et al., 2017). There was no effect of stimulation on mean heart rate or its *SD*.

A computation model confirmed that the peak electric field strength was over the rIFG. A finite element method (FEM) head model was created using Simnibs (Thielscher, Antunes, & Saturnino, 2015; Windhoff, Opitz, & Thielscher, 2013). This standard five compartment head model (white matter, grey matter, cerebrospinal fluid, skull, and skin) was further extended to include neck and shoulder parts. Conductivity values for various tissues were used as in (Opitz, Paulus, Will, & Thielscher, 2015). The electrode montage was modeled as described in the experimental section. Simulations of the tDCS electric field were performed using Simnibs version 2.0.1.

### 2.5 MRI acquisition

A T1 and fMRI sequences were acquired on a 3 T Siemens Verio (Siemens, Erlangen, Germany), using a 32‐channel head coil, with parameters similar to Violante et al. (2017). Functional MRI images were obtained using a T2*‐weighted gradient‐echo, echoplanar imaging (EPI) sequence, 3 mm^3^ isotropic voxel, repetition time (TR) 2 s, echo time (TE) 30 ms, flip angle (FA) 80°, field of view 192 × 192 × 105 mm, 64 × 64 matrix, 35 slices, GRAPPA acceleration factor = 2, run time of 12min 24 s. Standard T1‐weighted structural images were acquired using an MP‐RAGE sequence, 1 mm^3^ isotropic voxel, TR 2.3 s, TE 2.98 ms, inversion time 900 ms, FA 9°, field of view 256 × 256 mm, 256 × 256 matrix, 160 slices, GRAPPA acceleration factor = 2, run time of around 4.5min.

### 2.6 fMRI preprocessing

Data preprocessing was performed using the FMRI Expert Analysis Tool (FEAT) Version 6.00, from FMRIB's Software Library (FSL; Smith et al., 2004; Jenkinson, Beckmann, Behrens, Woolrich, & Smith, 2012). We performed motion correction using MCFLIRT (Jenkinson, Bannister, Brady, & Smith, 2002), removal of low‐frequency drifts (high‐pass filter of 0.01 Hz: set to well below the frequency of expected network changes), spatial smoothing (Gaussian kernel filter with a full width at half maximum of 6 mm), brain extraction to remove nonbrain tissue (BET; Smith, 2002), and co‐registration using FMRIB's Nonlinear Image Registration tool (FNIRT) to register the participant's fMRI volumes to Montreal Neurological Institute (MNI) 152 standard space using the T1‐weighted scan as an intermediate.

Single‐session ICA was performed for each run using Multivariate Exploratory Linear Optimized Decomposition (MELODIC; Beckmann et al., 2005). The resulting components were automatically classified into signal and noise using FMRIB's ICA‐based Xnoiseifier (FIX; Griffanti et al., 2015; Salimi‐khorshidi, Douaud, Beckmann, & Matthew, 2015). FIX was previously trained in an independent cohort of 20 individuals acquired in the same scanner with the same imaging parameters. Classifications were manually inspected and adjusted when required. Independent components classified as noise were subsequently removed from each voxel's time series.

### 2.7 fMRI analysis: activation

The tDCS–fMRI CRT was analyzed with FSL's FMRI Expert Analysis Tool (FEAT). Subject‐level general linear models were constructed with the following regressors of interest: [all task blocks], [all anodal blocks], [all cathodal blocks], an interactive regressor for [task + anodal], and an interactive regressor for [task + cathodal]. The implicit baseline was the [“rest” + sham] blocks, which was not modeled in the GLM. Using this GLM, the [all anodal blocks] and [all cathodal blocks] regressors demonstrate the main effects of anodal or cathodal stimulation in the absence of task. Using FSL's FEAT tool to create the interactive regressors of [task + anodal] and [task + cathodal] allowed us to demonstrate the interactive effects of anodal and cathodal tDCS and task. Regressors were created by convolving a boxcar kernel with a canonical double‐gamma hemodynamic response function. The GLM design matrices consisted of the regressors of interest and their first temporal derivatives, in order to more accurately model transitions in the hemodynamic response function at the onset and offset of each block. Six movement regressors to account for movement‐related noise, and a regressor of no interest for the periods of black screen, were also included. The interactive effects of anodal and cathodal tDCS and “rest” were investigated using a second GLM constructed in the same way, but substituting the [all task blocks] regressor with a regressor for [all “rest” blocks']. In this GLM, the interactive regressors of [“rest” + anodal] and [“rest” + cathodal] demonstrate the interactive effects of anodal and cathodal tDCS during “rest”.

A higher‐level mixed effects (FLAME 1 + 2) analysis of group effects was performed to combine all runs and all participants for the parameter estimates [task + anodal], [task + cathodal], [“rest” + anodal] and [“rest” + cathodal]. The inverse estimates were also run. A third‐level mixed effects (FLAME 1 + 2) analysis was performed to investigate: [“rest” + anodal] > [“rest” + cathodal] (and its inverse contrast) and [task + anodal] > [task + cathodal] (and its inverse contrast). The final *Z* statistical images were thresholded using a Gaussian random field‐based cluster inference with a height determined by a threshold of *Z* > 3.1 and a corrected cluster significance threshold of *p* = .05.

A region of interest (ROI) approach was taken to compare the effects of anodal and cathodal tDCS during different brain states (“rest” or task performance). Two ROIs were investigated: the “task activated” network and the “task deactivated” network. The “task activated” network was a binarized mask encompassing those regions showing increased BOLD activity in the contrast [task > “rest”] in the separate shorter CRT with no stimulation, and the “task deactivated” network was a binarized mask encompassing those regions showing decreased BOLD activity from the same contrast.

### 2.8 fMRI: functional connectivity analysis

Whole‐brain psychophysiological interaction (PPI) analyses (O'Reilly, Woolrich, Behrens, Smith, & Johansen‐Berg, 2012) were performed to assess the effect of tDCS and brain state on functional connectivity (FC). We used the following regions of interest: rIFG and dACC/pre‐SMA (forming the SN) and the posterior cingulate cortex (PCC) and ventromedial prefrontal cortex (vmPFC, forming the DMN).

Regions of interest were defined as follows:

- rIFG: this was generated using MANGO (Multi‐image Analysis GUI) software (http://ric.uthscsa.edu/mango/mango.html) by defining a 22.5 mm radius sphere centered on the center coordinates F8. This was obtained from the projection of the electrode position onto the cortical surface (Koessler et al., 2009) and converted to MNI space using the Nonlinear Yale MNI to Talairach Conversion Algorithm (Lacadie, Fulbright, Constable, & Papademetris, 2009; Lacadie et al., 2008) (F8: *x* = 55, *y* = 30, *z* = −1). The ROI was predominantly over the pars triangularis of the rIFG. Areas outside the cortex were removed by masking with an MNI brain mask from the FSL library.
- Dorsal ACC/pre‐SMA (dACC): generated by inflating a 10 mm sphere around the peak voxel in the anterior part of the increased BOLD activity from the contrast [task > “rest”] from the independent, non‐tDCS CRT.
- PCC: generated by inflating a 10 mm sphere around the peak voxel in the posterior part of the decreased BOLD activity from the contrast [task > “rest”] for the independent, non‐tDCS CRT.
- vmPFC: generated by inflating a 10 mm sphere around the peak voxel in the anterior part of the decreased BOLD activity from the contrast [task > “rest”] for the independent, non‐tDCS CRT.

Time‐courses for each region were extracted for each participant (the physiological term). Two subject‐level GLMs were run, with regressors as described above for the initial fMRI analysis. For each model, the physiological term and the psychological term were used to create the PPI interaction term, the remaining regressors were also included in the model. A generalized PPI model was used (McLaren, Ries, Xu, & Johnson, 2012). Bonferroni correction of the group result was used to correct for multiple comparisons across different ROIs.

## 3 RESULTS

### 3.1 Behavior

There were no significant effects of stimulation on behavioral performance. A three‐level anova with stimulation type as factors (sham/anodal/cathodal) did not show a main effect of stimulation on either CRT accuracy or any parameter of the exGaussian distribution for overall reaction times (all *F* < 1, all *p* > .05). The mean reaction times were: anodal = 506.9 ms, cathodal = 509.3 ms, and sham = 511.7 ms. There was also no consistent behavioral change over time (Supporting Information). The absence of a significant main effect of stimulation on behavior suggests that the interpretation of the subsequent neuroimaging results is not confounded by condition differences in CRT performance.

### 3.2 The effects of tDCS on brain activity are dependent on cognitive state

**Figure 2:**
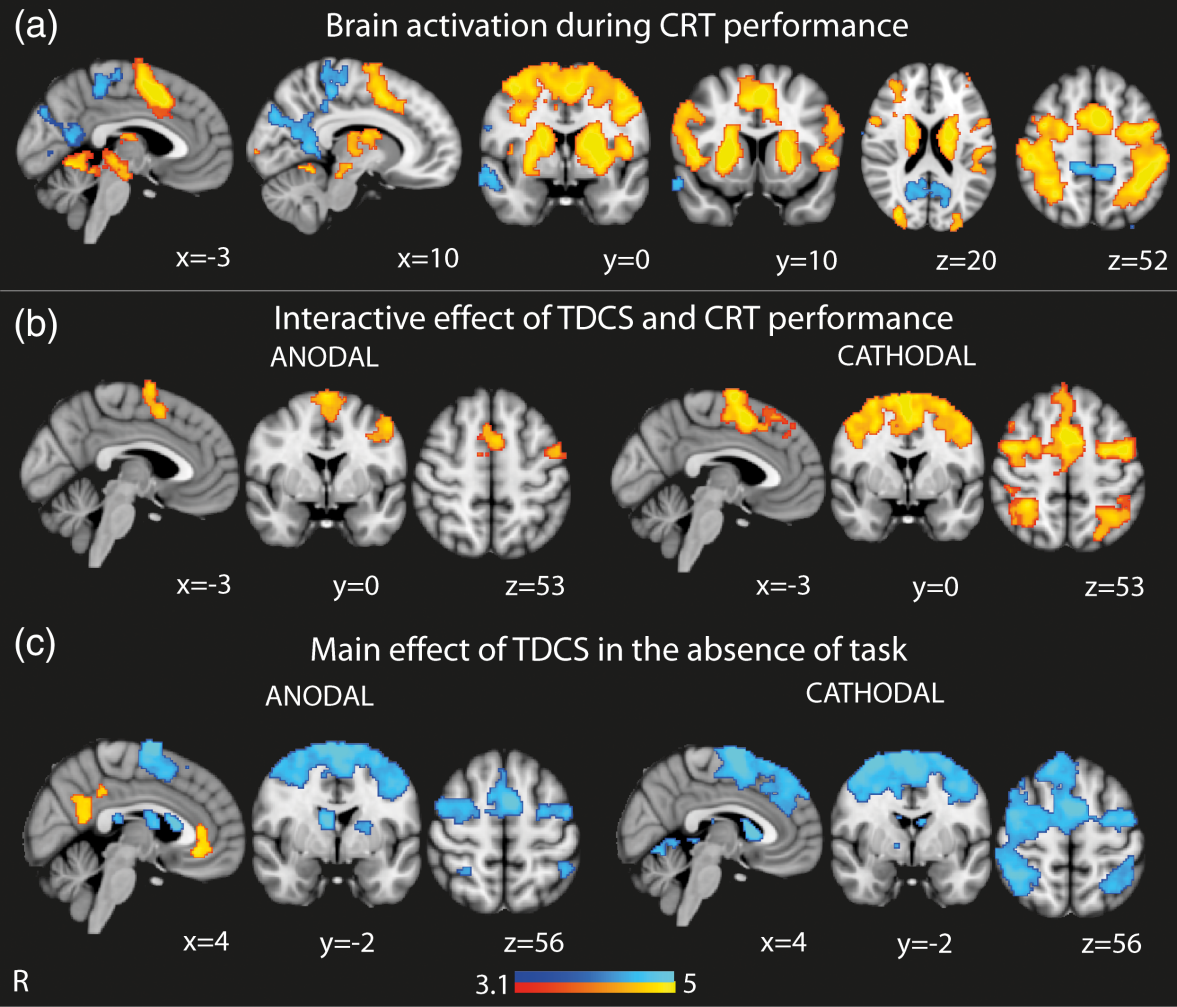
Brain activity with tDCS during “rest” and CRT performance. (a) Overlay of brain activation and deactivation associated with CRT performance (with no TDCS). (b) Brain areas showing greater activation when tDCS is applied during CRT performance. Results shown demonstrate the interactive effects of tDCS and CRT performance. (c) Brain areas showing greater activation and deactivation as a result of the main effect of tDCS in the absence of CRT performance. Warm colors represent brain regions showing more activation and cool colors represent brain regions showing more deactivation. Results are superimposed on the MNI152 1 mm brain template. Cluster corrected *z* = 3.1, *p* < .05 [Color figure can be viewed at https://wileyonlinelibrary.com]

Both anodal and cathodal tDCS produced widespread BOLD changes in brain areas anatomically remote from the cortical area being stimulated. TDCS accentuated the patterns of activation and deactivation normally observed in each cognitive state. CRT performance compared to rest was characterized by increased BOLD response within the FPCN, including the SN, as well as primary sensory/motor cortices, bilateral thalami, and basal ganglia, and decreased BOLD signal within the DMN (Figure 2a). The interactive effects of both anodal and cathodal stimulation applied during CRT performance were increased activity within the dorsal anterior cingulate cortex/presupplementary motor area (dACC/pre‐SMA) and lateral prefrontal regions. Cathodal tDCS was also associated with an additional increased BOLD response within the SN, including the rIFG, as well as additional increases in bilateral frontal eye fields, bilateral occipital and superior parietal regions (Figure 2b). There were no areas of decreased BOLD response, compared with CRT performance alone.

In contrast, the main effect of tDCS (in the absence of task because task effects were modeled in the interaction term) was to accentuate deactivation of the dACC/pre‐SMA (Figure 2c). Cathodal tDCS was associated with extensive deactivation in the rIFG and underlying anterior insula, bilateral superior frontal gyri, bilateral frontal eye fields, and bilateral superior parietal regions. Anodal tDCS was additionally associated with activation within the DMN, with increased activity of the PCC, the precuneus, and the vmPFC; (Figure 2c).

**Figure 3:**
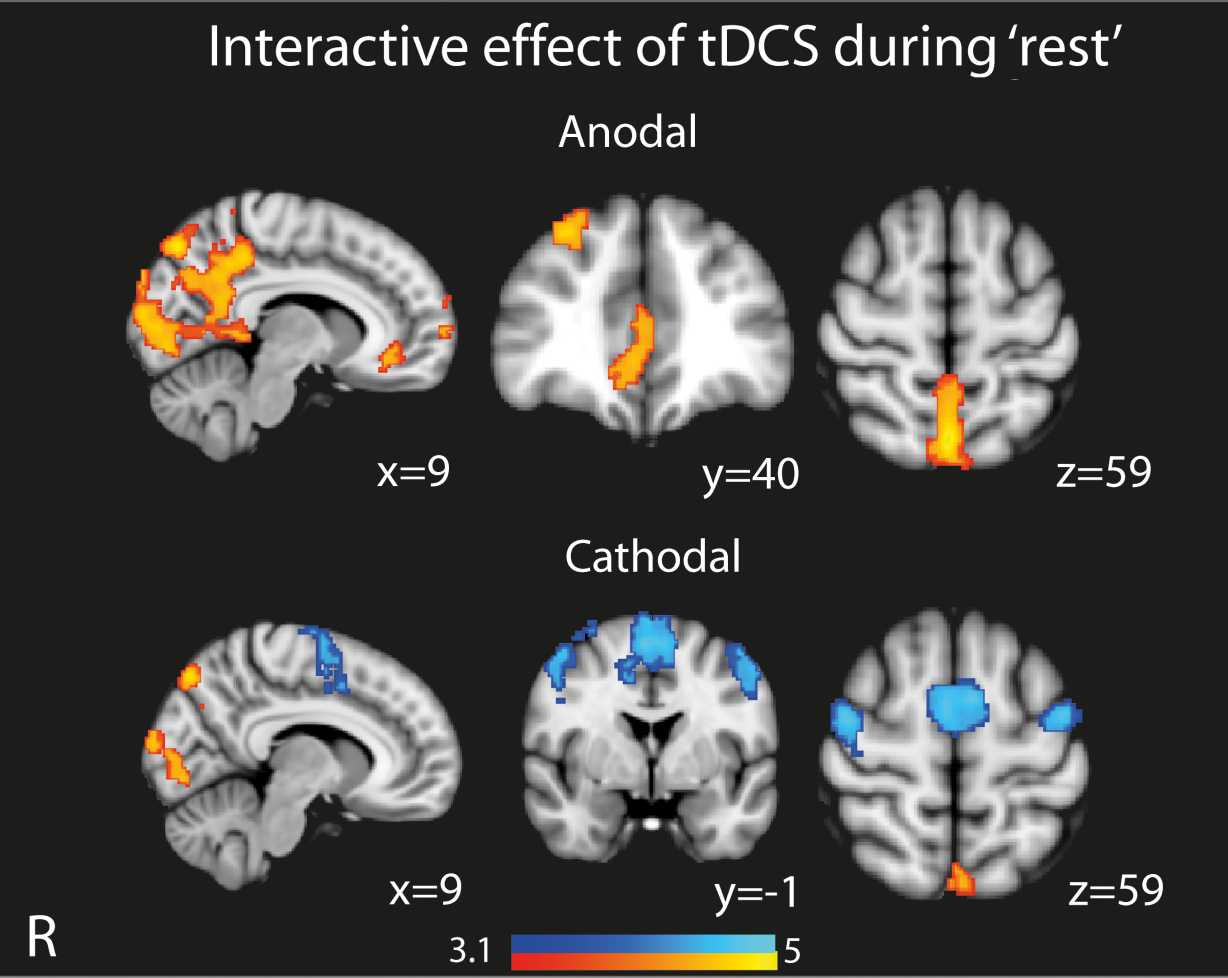
Overlay of brain areas showing activation and deactivation when tDCS is applied during “rest.” Results shown demonstrate the interactive effects of tDCS and “rest.” Results are superimposed on the MNI152 1 mm brain template. Cluster corrected z = 3.1, p < .05 [Color figure can be viewed at https://wileyonlinelibrary.com]

To clarify the effect of tDCS during “rest,” we used a second GLM that specifically modeled the interaction between stimulation and rest (Figure 3). This showed that anodal tDCS applied during “rest” produced increased activation within the DMN, including the vmPFC, as well as medial occipital regions. Conversely, cathodal tDCS produced less widespread activation of the DMN, as well as deactivation of SN and FPCN areas. These findings are similar to the main effect of tDCS in the absence of task as described above (Figure 2c), confirming that tDCS is modulating activity during rest in a distinct way to its effect during task performance.

### 3.3 Polarity‐dependent effects of tDCS interact with underlying brain state

**Figure 4:**
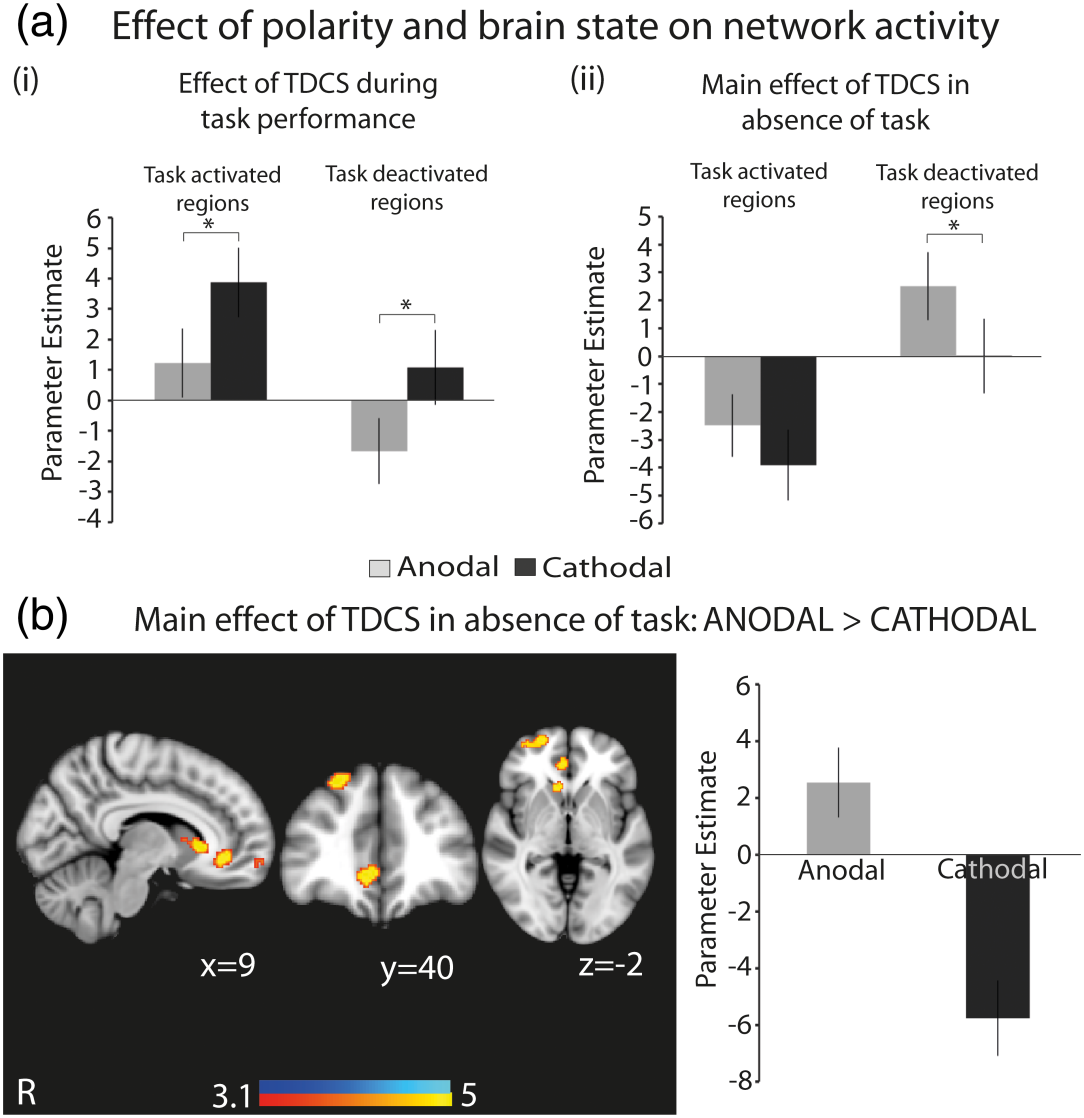
An interaction between polarity and brain state on network activation. (a) Graphs showing changes in BOLD activity in task‐related networks under anodal and cathodal tDCS during (i) “rest” and (ii) CRT performance. “Task‐activated regions” refers to the areas showing increased activation, and “task‐deactivated regions” refers to areas showing decreased activation, during CRT performance, as shown in Figure 2a. (b) Overlay of brain areas showing greater activation with anodal tDCS, compared with cathodal tDCS, in the absence of task. The accompanying bar chart shows mean activation in the overlaid regions under anodal and cathodal tDCS, and is provided to demonstrate the increase in BOLD activity under anodal tDCS and decrease in BOLD activity under cathodal tDCS. Results are superimposed on the MNI152 1 mm brain template. Cluster corrected *z* = 3.1, *p* < .05. Bar charts denote mean values, error bars are *SEM*s, * denotes *p* < .05 [Color figure can be viewed at https://wileyonlinelibrary.com]

The effects of tDCS were dependent on the polarity of stimulation (Figure 4). A targeted analysis specifically comparing the effects of stimulation within regions activated or deactivated by task showed polarity‐dependent effects. Both anodal and cathodal tDCS modulated BOLD activity in the same direction but did so to different degrees depending on the brain state (Figure 4a). During task performance, cathodal tDCS produced greater activation of task‐activated regions compared to anodal tDCS, while anodal tDCS produced greater deactivation in task‐deactivated regions compared to cathodal tDCS (Figure 4a‐i).

Conversely, the main effect of anodal tDCS in the absence of task was to increase activation within task‐deactivated regions, whereas cathodal tDCS did not (Figure 4a‐ii). The whole‐brain contrast of anodal and cathodal stimulation confirmed a similar effect, with anodal tDCS increasing activity in the right vmFPC and superior frontal gyrus, whereas cathodal tDCS decreased BOLD activity in the same regions (Figure 4b).

### 3.4 tDCS modulates SN and DMN connectivity

We next investigated whether tDCS modulated the FC of the SN, and DMN, using whole‐brain PPI analysis (Friston et al., 1997; O'Reilly et al., 2012). We interrogated the connectivity between key nodes of the SN (the rIFG and dACC/pre‐SMA) and DMN (the vmPFC and PCC) and the rest of the brain.

#### 3.4.1 Changes in FC of SN nodes

**Figure 5:**
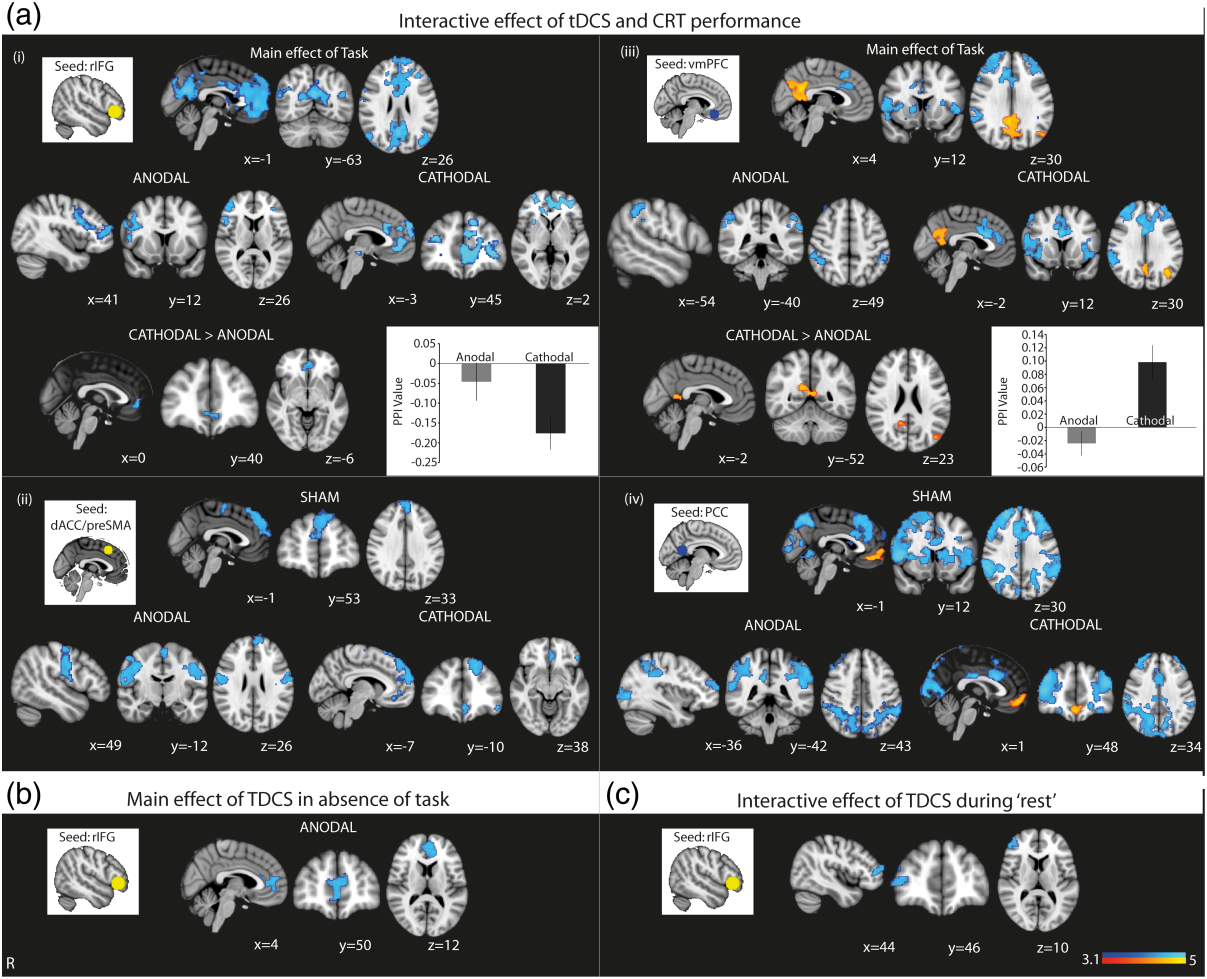
Functional connectivity during different stimulation polarities and brain states. (a) Results show increased (warm colors) and decreased (cool colors) FC between the overlaid areas and the (i) right inferior frontal gyrus (rIFG), (ii) dorsal anterior cingulate cortex (dACC), (iii) ventromedial prefrontal cortex (vmPFC), and (iv) the posterior cingulate cortex (PCC) when stimulation is applied during CRT performance. Results for anodal and cathodal tDCS denote the change in FC over and above that produced by CRT performance in the sham condition. Accompanying insets show the seed ROI used for the PPI analysis. Where there is a significant whole‐brain result for the direct comparison of anodal and cathodal stimulation, for the rIFG and vmPFC seeds, this is also shown, in (i) and (iii). Accompanying bar chart shows the mean PPI value in the overlaid areas under anodal and cathodal tDCS, and are provided to show the direction of change. (b) Results show decreased (cool colors) FC between the overlaid areas and the rIFG for the main effect of anodal tDCS. (c) Results show decreased (cool colors) FC between the overlaid areas and the rIFG when anodal tDCS is applied during “rest”. There were no significant whole‐brain results from the other seed regions. Results are superimposed on the MNI152 1 mm brain template. Cluster corrected *z* = 3.1, *p* < .05. Bar charts denote mean values, error bars are *SEM*s [Color figure can be viewed at https://wileyonlinelibrary.com]

CRT performance reduced FC between the rIFG, the site of stimulation, and areas comprising the DMN. Anodal tDCS further decreased the FC between the rIFG and middle frontal gyrus, as well as within the rIFG. Cathodal tDCS further decreased the FC between the rIFG and vmPFC and anterior paracingulate gyrus. A direct comparison of the effects of anodal and cathodal tDCS showed that cathodal tDCS produced a greater decrease in FC between the rIFG and the vmPFC (Figure 5a‐i). CRT performance also reduced FC between the dACC/pre‐SMA and the superior frontal gyrus. Anodal tDCS further decreased FC between the dACC/pre‐SMA and the superior frontal gyrus and bilateral primary motor cortices, and cathodal tDCS further decreased FC between the dACC/pre‐SMA and the superior frontal gyrus and vmPFC (Figure 5a‐ii,b).

#### 3.4.2 Changes in FC of DMN nodes

Stimulation produced marked effects on the FC of the DMN. CRT performance increased FC between the two key nodes of the DMN, the vmPFC, and PCC. Concurrently, CRT performance decreased FC between the vmPFC and dACC/pre‐SMA and bilateral inferior frontal regions. Anodal tDCS further decreased FC between the vmPFC and bilateral superior parietal regions. Cathodal tDCS further decreased FC between the vmPFC and the dACC and bilateral inferior frontal regions, as well as accentuating the increase in within‐DMN connectivity seen during task alone. A direct comparison of the effects of anodal and cathodal tDCS showed that cathodal tDCS increased the FC between the vmPFC and the PCC, whereas anodal tDCS did not (Figure 5a‐iii,b). Stimulation effects were also seen on PCC connectivity. CRT performance increased FC between the PCC and vmPFC and decreased FC between the PCC and the FPCN as well as bilateral caudate. Anodal tDCS further decreased FC between the PCC and the frontal poles and bilateral occipital and superior parietal regions. Cathodal tDCS further increased FC between the PCC and vmPFC and decreased FC between the PCC and dACC/pre‐SMA, bilateral occipital and superior parietal regions. (Figure 4a‐iv).

The main effect of anodal tDCS in the absence of task was decreased FC between the rIFG and the vmPFC, but there was no other significant main effect of tDCS on FC (Figure 5b). Similar analyses were performed using the second GLM that specifically modeled the interaction between stimulation and rest, which showed decreased rIFG connectivity with right frontal regions (Figure 5c). There were no other significant effects of stimulation during rest on FC.

There were no correlations between tDCS‐related changes in connectivity and BOLD activity.

### 3.5 Subjective experience

Prior to having combined tDCS and MRI, participants received two blocks of anodal and cathodal tDCS (15 s each) in a randomized, blind order. Participants were asked to rate their sensation of “itching,” “pain,” “metallic taste,” “burning,” “anxiety,” and “tingling” on a scale of 1–5 (1 = nil, 2 = mild, 3 = moderate, 4 = strong, 5 = unbearable). There were no differences observed between average ratings given to anodal versus cathodal tDCS on any category.

## 4 DISCUSSION

We show that widespread modulation of the activity and connectivity of networks involved in cognitive control is achievable with tDCS, of even brief durations, applied to the right inferior frontal gyrus. The network modulation produced by tDCS is dependent on both the underlying state of the brain network and the polarity of stimulation. We show an interaction between tDCS polarity and brain state on network activity, such that the same polarity tDCS caused distinct effects on the brain depending on whether subjects were engaged in a cognitive task or not. These findings are important both for interpreting previous studies, potentially explaining the variability of tDCS effects, and also for shaping the design of future stimulation studies.

Previous tDCS studies have mainly focused on the effects of stimulation on motor cortex, concluding that anodal tDCS increased neuronal excitability and cathodal tDCS reduced it (Purpura & Mcmurtry, 1965) (Nitsche & Paulus, 2000; Stagg & Nitsche, 2011). However, it is unclear whether these results can be extrapolated to other parts of the cortex. Many cognitive studies have investigated the effects of tDCS on behavior. However, relatively few (~20%) have shown the expected distinction between anodal and cathodal stimulation (Jacobson, Koslowsky, & Lavidor, 2012). Only a small number of studies have used concurrent tDCS/fMRI to directly investigate physiological effects, and these have shown that anodal and cathodal tDCS are both capable of producing increases and decreases in cortical activity and connectivity (Amadi et al., 2014; Antal et al., 2011; Polanía, Paulus, & Nitsche, 2012). Our study extends this work by showing that both anodal and cathodal tDCS can produce increases and decreases in the activity and FC of cognitive networks, but that the effects of tDCS polarity are modulated by the state of the network when it is stimulated. This further demonstrates that the effect of polarity is more nuanced than a simple dichotomy where anodal stimulation produces excitation of cortical activity and cathodal stimulation is inhibitory.

### 4.1 Stimulation of the right inferior frontal gyrus modulates activity and connectivity in large‐scale cognitive networks

Brief stimulation of a single brain region, the rIFG, modulated activity in remote parts of two large‐scale brain networks involved in cognitive control, the DMN, and SN, as well as modulating FC between them. The rIFG acts as a hub connecting many other cortical regions and is activated by a wide range of cognitive functions (Sporns, 2013). It is thought to coordinate changes in activity across other cognitive control networks when switching between different task states (Duncan & Owen, 2000; Fedorenko, Duncan, & Kanwisher, 2013; Hampshire & Sharp, 2015; Sridharan et al., 2008). Hence, our results may reflect widespread network changes in brain regions remote to, but connected to the rIFG, potentially accentuating the control mechanism exerted by the rIFG.

We are unaware of any previous tDCS–fMRI study investigating the effects of rIFG stimulation. A small number of tDCS–fMRI studies applying tDCS to the primary motor cortex (M1) or dorsolateral prefrontal cortices (dlPFC) have shown effects distant from the site of stimulation (Antal et al., 2011; Park et al., 2013; Peña‐Gómez et al., 2012; Polania et al., 2012; Sehm et al., 2013; Stagg & Johansen‐berg, 2013). In addition, a study comparing M1 and left dorsolateral prefrontal cortex tDCS found that M1 tDCS modulated connectivity of sensorimotor networks, while tDCS to the DLPFC additionally modulated effective networks (Sankarasubramanian et al., 2017). A previous study stimulating the left IFG found that anodal and cathodal left IFG stimulation differentially modulates brain activation during a verbal frequency task, though this was assessed with functional near infra‐red spectroscopy so did not assess changes in a distributed network (Ehlis, Haeussinger, Gastel, Fallgatter, & Plewnia, 2016). Another study of left IFG tDCS found that anodal tDCS had remote effects in both resting‐state fMRI and during a word retrieval task (Meinzer et al., 2015). Our study substantially extends the literature by investigating the remote effects of tDCS and its interaction with the task for networks specifically involved in cognitive control.

### 4.2 Short durations of stimulation produced large physiological effects

Large changes in BOLD activity were seen after seconds, rather than minutes, of stimulation. This is compatible with in vitro animal studies that show applying cortical surface currents cause immediate changes in evoked potentials and spontaneous spike activity changes (Purpura & Mcmurtry, 1965), and human studies showing that 4 s of tDCS can produce changes in M1 excitability (Nitsche & Paulus, 2000). Our study extends these findings by showing that rapid changes in the activity of cognitive brain networks are possible with short durations of tDCS. tDCS has been shown to induce Ca^2+^ waves in astrocytes within seconds of application, suggesting that nonneuronal mechanisms might contribute to early neurobiological effects (Monai et al., 2016). However, purely nonneuronal mechanisms, such as the direct effects of tDCS on brain hemodynamics, would be unlikely to produce the interaction of stimulation with a task that we observed. This suggests that the effects of tDCS observed in our study reflect, at least in part, direct neuronal or astrocytic effects.

### 4.3 The effects of stimulation on cognitive network activity depends on brain state

Our results also demonstrate that the physiological effects of tDCS are contingent on the current activity in that network. Distinct effects of the same polarity of tDCS were seen in a given network, depending on the cognitive state. This converges with animal work demonstrating that tDCS does not directly cause action potentials, but instead alters the probability of their occurrence (Purpura & Mcmurtry, 1965) (Stagg & Nitsche, 2011). Therefore, one would expect the effects of stimulation to vary depending on the populations of neurons active at that time.

A relationship between cognitive brain state and stimulation effects is also suggested by the small number of behavioral studies which demonstrate that manipulations of task difficulty can influence the behavioral modulations seen with tDCS (Gill, Shah‐Basak, & Hamilton, 2015; Jones & Berryhill, 2012; Li et al., 2015; Sandrini, Fertonani, Cohen, & Miniussi, 2012; Wu et al., 2014). Transcranial alternating current stimulation also shows effects on cortical network activity and connectivity that are dependent on the cognitive brain state (Neuling, Rach, Herrmann, & Schwiedrzik, 2013; Violante et al., 2017; Vosskuhl, Huster, & Herrmann, 2015). The link between the effects of stimulation and brain state has particularly important implications for clinical studies, since tDCS may produce distinct effects depending on whether it is applied during an active task or rest. Hence, attempts to use tDCS to enhance cognitive rehabilitation will need to carefully control the behavior of a patient at the time of stimulation.

### 4.4 The effects of stimulation polarity on cognitive network activity interact with underlying brain state

Our study demonstrates an interaction of underlying brain state and the polarity of stimulation on network activity. A similar interaction between task state and motor cortex excitability has been seen before, as assessed by motor evoked potential (MEP) size. MEP size was increased when anodal tDCS was given at rest but was decreased if applied during the task (Antal, Terney, Poreisz, & Paulus, 2007) However, to the best of our knowledge, this interaction between brain state and tDCS polarity has not been seen before in cognitive networks.

Current theories of how tDCS acts at the cellular level do not provide a simple explanation for this result. At the synaptic level, there is evidence that anodal and cathodal tDCS can have distinct effects on neurotransmitter levels. For example, the effects of anodal, but not cathodal tDCS, are abolished by NMDA_R_, voltage‐gated Ca^2+^ and Na^+^ receptor blockade (Stagg & Nitsche, 2011). Additionally, a small number of studies have found that anodal tDCS decreases local GABA concentration and increases local Glutamine concentration, whereas cathodal tDCS decreases local Glutamine concentration (Hunter et al., 2015; Kim, Stephenson, Morris, & Jackson, 2014; Stagg et al., 2009). These changes could underlie the observed, and differential, effects of tDCS on local excitatory and inhibitory circuits (Sasaki, Miyaguchi, Kotan, Kojima, & Kirimoto, 2016; Stagg & Nitsche, 2011; Tazoe, Endoh, Kitamura, & Ogata, 2014; Wiethoff, Hamada, & Rothwell, 2014). As local changes in the excitatory/inhibitory balance are thought to produce changes in large‐scale brain networks (Deco, Hagmann, Romani, Mantini, & Corbetta, 2014), this might provide a mechanism for the remote effects on network activity we observed. However, we show that both cathodal and anodal tDCS caused a change of BOLD response in the same direction relative to baseline, which cannot be explained by opposing effects on excitatory and inhibitory neurotransmitter levels.

Our results might be explained by a complex interaction between stimulation, cellular structure, and orientation. in vitro and modelling studies demonstrate that the effect of tDCS on soma and dendrite polarization is influenced by neuronal shape, cortical layer, and the orientation of neuronal processes in the electrical field (Arlotti, Rahman, Minhas, & Bikson, 2012; Lafon, Rahman, Bikson, & Parra, 2017; Radman, Ramos, Brumberg, & Bikson, 2009; Rahman et al., 2013). A cortical region that is activated by a task will include subpopulations of neurons, some excitatory and some inhibitory, with different morphologies, orientations and occupying different cortical layers. As a result, different polarities of tDCS, which can really be considered as different directions of current flow, may activate different subpopulations of neurons within the same region. In addition, there is a complex relationship between alterations in excitatory/inhibitory balance and BOLD activity mediated by alterations in blood oxygenation and flow (Logothetis, Pauls, Augath, Trinath, & Oeltermann, 2001). For example, increased BOLD signal can increase secondary to activity of both excitatory and inhibitory circuits or increased activity of excitatory circuits with decreased activity of inhibitory circuits. Hence, an interaction between brain state and polarity may arise due to different subpopulations being activated under different combinations of task and polarity.

The differential effects of tDCS on neurons of different orientations also limit how much the effect of tDCS on cognitive networks can be predicted through extrapolating from results of motor cortex studies. Such studies use montages very different to ours, resulting in different patterns of current flow, and different electrical fields along the somatodendritic axis of neurons. Careful modeling studies, incorporating neuronal subpopulations, combined with in vivo electrophysiological measurements, would be informative in clarifying the interaction between polarity and neuronal orientation.

### 4.5 Right inferior frontal gyrus stimulation modulates SN and DMN connectivity

Large changes in FC were produced by stimulation of the right IFG. Both cathodal and anodal tDCS applied during task performance modulated FC within the DMN, and also changed interactions between the SN and the DMN. Previous studies have found that within‐DMN connectivity is increased in cognitive tasks (Hampson, Driesen, Skudlarski, Gore, & Constable, 2006; Vatansever, Manktelow, Sahakian, Menon, & Stamatakis, 2017; Vatansever, Menon, Manktelow, Sahakian, & Stamatakis, 2015) and we observed a similar change in the interaction of the two main nodes of the DMN (the PCC and vmPFC) during task performance. Previous studies have also shown that connectivity between the SN and DMN is correlated with cognitive task performance, with the SN having a causal effect on DMN activity (Chiong et al., 2013; Jilka et al., 2014; Sridharan et al., 2008). This suggests that while our study was not set up to address behavioral modulation, the effects of tDCS on cognitive network connectivity which we demonstrate could have behavioral relevance. Future tDCS behavioral studies should concurrently assess network connectivity changes, in order to determine the extent to which behavioral modulation by tDCS can be explained by modulation of network connectivity.

Stimulation‐induced changes in FC also showed marked polarity specificity. Cathodal tDCS decreased FC between the rIFG node of the SN and vmPFC node of the DMN and concurrently increased FC between the vmPFC and the rest of the DMN, effects not seen with anodal tDCS. This suggests that cathodal tDCS is capable of modulating interactions between and within intrinsic connectivity networks, with changes in rIFG‐vmPFC connectivity being particularly prominent. Outside of the cognitive field, abnormal patterns of vmPFC activity has been implicated in the development of depression (Koenigs & Grafman, 2009) and studies of brain stimulation in depression suggest that cerebral blood flow of the vmPFC is an important predictor for clinical response (Kito, Hasegawa, & Koga, 2012a, 2012b). Future research might investigate whether cathodal tDCS is capable of affecting emotional state.

### 4.6 Limitations

We focused on rIFG stimulation and did not investigate the effects of stimulating other parts of the cognitive control system. Therefore, we cannot comment on whether the results we have seen are specific to the rIFG. Stimulation of other highly connected areas may also produce similar widespread network effects. Network hierarchy analyses, comparing multiple different tDCS targets, would be a sensible approach to test this hypothesis.

Our experimental design does not permit exploration of tDCS effects that may have persisted after the end of the stimulation. Studies of motor cortex suggest that the intra‐stimulation and poststimulation effects of tDCS on cortical excitability are different (Nitsche et al., 2005; Nitsche & Paulus, 2000), which may also extrapolate to cognitive tDCS. It is uncertain how long poststimulation effects last for so we cannot guarantee that there were no carryover effects. There have been no studies formally investigating the stimulation duration required to produce after‐effects in the cognitive realm. However, in the motor cortex, 3 min of stimulation appears to be the minimum duration to produce after‐effects (Nitsche & Paulus, 2000). Therefore, we think that our results are likely to be free from significant bias as a result of any potential carryover, especially since we also pseudorandomized the order of stimulation such that any carryover should only add noise to the data rather than systematic bias. Additionally, our fMRI analysis looked at the effects of stimulation + task or stimulation + “rest” over and above the effects of sham + task and sham + “rest”, so any contamination of the sham block by active stimulation is more likely to cause a false negative than a false positive result. Nevertheless, the relationship between stimulation duration and effect is important to understand. Dosing studies, particularly of non‐M1 areas, will help to clarify the relationship between duration of stimulation and effects, particularly as duration may also interact with brain state and polarity.

## 5 CONCLUSION

The implications from our study are far‐reaching. We demonstrate that widespread modulation of cognitive networks is achievable with tDCS and that this effect is highly dependent on the underlying brain network state and polarity. The effect of stimulation, therefore, is an emergent property of the applied current in combination with the underlying brain state. Our results suggest many avenues for future investigations and have important implications for the translation of tDCS for clinical use. Our study strongly argues for the need for concurrent neurobiological assessment in cognitive tDCS studies and supports a new paradigm for investigating the neurophysiological effects of stimulation.

## References

[hbm24420-bib-0001] Amadi, U. , Ilie, A. , Johansen‐Berg, H. , & Stagg, C. J. (2014). Polarity‐specific effects of motor transcranial direct current stimulation on fMRI resting state networks. NeuroImage, 88, 155–161. 24287440 10.1016/j.neuroimage.2013.11.037 PMC3991849

[hbm24420-bib-0002] Antal, A. , Polania, R. , Schmidt‐Samoa, C. , Dechent, P. , & Paulus, W. (2011). Transcranial direct current stimulation over the primary motor cortex during fMRI. NeuroImage, 55(2), 590–596. 21211569 10.1016/j.neuroimage.2010.11.085

[hbm24420-bib-0003] Antal, A. , Terney, D. , Poreisz, C. , & Paulus, W. (2007). Towards unravelling task‐related modulations of neuroplastic changes induced in the human motor cortex. The European Journal of Neuroscience, 26(9), 2687–2691. 17970738 10.1111/j.1460-9568.2007.05896.x

[hbm24420-bib-0004] Arlotti, M. , Rahman, A. , Minhas, P. and Bikson, M. (2012). *Axon terminal polarization induced by weak uniform DC electric fields: A modeling study*. Proceedings of the Annual International Conference of the IEEE Engineering in Medicine and Biology Society (EMBS, pp. 4575–4578), vol. 2. 10.1109/EMBC.2012.6346985 23366946

[hbm24420-bib-0005] Beckmann, C. F. , Deluca, M. , Devlin, J. T. , Smith, S. M. , Hospital, J. R. , & Ox, O. (2005). Investigations into resting‐state connectivity using independent component analysis. Philosophical Transactions of the Royal Society B: Biological Sciences, 360, 1001–1013. 10.1098/rstb.2005.1634 PMC1854918 16087444

[hbm24420-bib-0081] Brainard, D. H. (1997). The psychophysics toolbox. Spatial Vision, 10, 433–436. 9176952

[hbm24420-bib-0006] Bressler, S. L. , & Menon, V. (2010). Large‐scale brain networks in cognition: Emerging methods and principles. Trends in Cognitive Sciences, 14(6), 277–290. 20493761 10.1016/j.tics.2010.04.004

[hbm24420-bib-0007] Buckner, R. L. , Andrews‐Hanna, J. R. , & Schacter, D. L. (2008). The brain's default network: Anatomy, function, and relevance to disease. Annals of the New York Academy of Sciences, 1124, 1–38. 18400922 10.1196/annals.1440.011

[hbm24420-bib-0008] Chiong, W. , Wilson, S. M. , D'Esposito, M. , Kayser, A. S. , Grossman, S. N. , Poorzand, P. , … Rankin, K. P. (2013). The salience network causally influences default mode network activity during moral reasoning. Brain, 136(6), 1929–1941. 23576128 10.1093/brain/awt066 PMC3673466

[hbm24420-bib-0009] Coffman, B. A. , Clark, V. P. , & Parasuraman, R. (2014). Battery powered thought: Enhancement of attention, learning, and memory in healthy adults using transcranial direct current stimulation. NeuroImage, 85, 895–908. 23933040 10.1016/j.neuroimage.2013.07.083

[hbm24420-bib-0010] Deco, G. , Hagmann, P. , Romani, G. L. , Mantini, D. , & Corbetta, M. (2014). How local excitation—Inhibition ratio impacts the whole brain dynamics. The Journal of Neuroscience, 34(23), 7886–7898. 24899711 10.1523/JNEUROSCI.5068-13.2014 PMC4044249

[hbm24420-bib-0011] Duncan, J. , & Owen, A. M. (2000). Common regions of the human frontal lobe recruited by diverse cognitive demands. Trends in Neurosciences, 23(10), 475–483. 11006464 10.1016/s0166-2236(00)01633-7

[hbm24420-bib-0012] Ehlis, A. , Haeussinger, F. B. , Gastel, A. , Fallgatter, A. J. , & Plewnia, C. (2016). Task‐dependent and polarity‐specific effects of prefrontal transcranial direct current stimulation on cortical activation during word fluency. NeuroImage, 140, 134–140. 26748077 10.1016/j.neuroimage.2015.12.047

[hbm24420-bib-0013] Fedorenko, E. , Duncan, J. , & Kanwisher, N. (2013). Broad domain generality in focal regions of frontal and parietal cortex. Proceedings of the National Academy of Sciences of the United States of America, 110(41), 16616–16621. 24062451 10.1073/pnas.1315235110 PMC3799302

[hbm24420-bib-0014] Fox, M. D. , Snyder, A. Z. , Vincent, J. L. , Corbetta, M. , Van Essen, D. C. , & Raichle, M. E. (2005). The human brain is intrinsically organized into dynamic, anticorrelated functional networks. Proceedings of the National Academy of Sciences of the United States of America, 102(27), 9673–9678. 15976020 10.1073/pnas.0504136102 PMC1157105

[hbm24420-bib-0015] Friston, K. J. , Buechel, C. , Fink, G. R. , Morris, J. , Rolls, E. , & Dolan, R. J. (1997). Psychophysiological and modulatory interactions in neuroimaging. NeuroImage, 6(3), 218–229. 9344826 10.1006/nimg.1997.0291

[hbm24420-bib-0016] Gill, J. , Shah‐Basak, P. P. , & Hamilton, R. (2015). It's the thought that counts: Examining the task‐dependent effects of transcranial direct current stimulation on executive function. Brain Stimulation, 8(2), 253–259. 25465291 10.1016/j.brs.2014.10.018

[hbm24420-bib-0017] Griffanti, L. , Salimi‐khorshidi, G. , Beckmann, C. F. , Auerbach, J. , Douaud, G. , Sexton, C. E. , … Smith, S. M. (2015). ICA‐based artefact removal and accelerated fMRI acquisition for improved resting state network imaging. NeuroImage, 95, 232–247. 10.1016/j.neuroimage.2014.03.034 PMC4154346 24657355

[hbm24420-bib-0018] Hampshire, A. , & Sharp, D. J. (2015). Contrasting network and modular perspectives on inhibitory control. Trends in Cognitive Sciences, 19(8), 445–452. 26160027 10.1016/j.tics.2015.06.006

[hbm24420-bib-0019] Hampson, M. , Driesen, N. R. , Skudlarski, P. , Gore, J. C. , & Constable, R. T. (2006). Brain connectivity related to working memory performance. The Journal of Neuroscience, 26(51), 13338–13343. 17182784 10.1523/JNEUROSCI.3408-06.2006 PMC2677699

[hbm24420-bib-0020] Horvath, J. C. , Forte, J. D. , Carter, O. , & Horvath, J. C. (2015). Quantitative review finds no evidence of cognitive effects in healthy populations from single‐session transcranial direct current stimulation (tDCS). Brain Stimulation, 8, 535–550. 25701175 10.1016/j.brs.2015.01.400

[hbm24420-bib-0021] Hunter, M. A. , Coffman, B. A. , Gasparovic, C. , Calhoun, V. D. , Trumbo, M. C. , & Clark, V. P. (2015). Baseline effects of transcranial direct current stimulation on glutamatergic neurotransmission and large‐scale network connectivity. Brain Research, 1594, 92–107. 25312829 10.1016/j.brainres.2014.09.066 PMC4358793

[hbm24420-bib-0022] Jacobson, L. , Koslowsky, M. , & Lavidor, M. (2012). tDCS polarity effects in motor and cognitive domains: A meta‐analytical review. Experimental Brain Research, 216, 1–10. 21989847 10.1007/s00221-011-2891-9

[hbm24420-bib-0023] Jenkinson, M. , Bannister, P. , Brady, M. , & Smith, S. (2002). Improved optimization for the robust and accurate linear registration and motion correction of brain images. NeuroImage, 841, 825–841. 10.1016/s1053-8119(02)91132-8 12377157

[hbm24420-bib-0024] Jenkinson, M. , Beckmann, C. F. , Behrens, T. E. J. , Woolrich, M. W. , & Smith, S. M. (2012). FSL. NeuroImage, 62, 782–790. 21979382 10.1016/j.neuroimage.2011.09.015

[hbm24420-bib-0025] Jilka, S. R. , Scott, G. , Ham, T. , Pickering, A. , Bonnelle, V. , Braga, R. M. , … Sharp, D. J. (2014). Damage to the salience network and interactions with the default mode network. The Journal of Neuroscience, 34(33), 10798–10807. 25122883 10.1523/JNEUROSCI.0518-14.2014 PMC4131006

[hbm24420-bib-0026] Jones, K. T. , & Berryhill, M. E. (2012). Parietal contributions to visual working memory depend on task difficulty. Frontiers in Psychiatry, 3, 1–11. 22973241 10.3389/fpsyt.2012.00081 PMC3437464

[hbm24420-bib-0027] Kim, S. , Stephenson, M. C. , Morris, P. G. , & Jackson, S. R. (2014). tDCS‐induced alterations in GABA concentration within primary motor cortex predict motor learning and motor memory: A 7T magnetic resonance spectroscopy study. NeuroImage, 99C, 237–243. 10.1016/j.neuroimage.2014.05.070 PMC4121086 24904994

[hbm24420-bib-0028] Kito, S. , Hasegawa, T. , & Koga, Y. (2012a). Cerebral blood flow in the ventromedial prefrontal cortex correlates with treatment response to low‐frequency right prefrontal repetitive transcranial magnetic stimulation in the treatment of depression. Psychiatry and Clinical Neurosciences, 66(2), 138–145. 22353326 10.1111/j.1440-1819.2011.02312.x

[hbm24420-bib-0029] Kito, S. , Hasegawa, T. , & Koga, Y. (2012b). Cerebral blood flow ratio of the dorsolateral prefrontal cortex to the ventromedial prefrontal cortex as a potential predictor of treatment response to transcranial magnetic stimulation in depression. Brain Stimulation, 5(4), 547–553. 22019081 10.1016/j.brs.2011.09.004

[hbm24420-bib-0030] Koenigs, M. , & Grafman, J. (2009). The functional neuroanatomy of depression: Distinct roles for the ventromedial and dorsolateral prefrontal cortex. Behavioural Brain Research, 6(2), 247–253. 10.1016/j.bbr.2009.03.004 PMC2680780 19428640

[hbm24420-bib-0031] Koessler, L. , Maillard, L. , Benhadid, A. , Vignal, J. P. , Felblinger, J. , Vespignani, H. , & Braun, M. (2009). Automated cortical projection of EEG sensors: Anatomical correlation via the international 10‐10 system. NeuroImage, 46(1), 64–72. 19233295 10.1016/j.neuroimage.2009.02.006

[hbm24420-bib-0082] Lacadie, C. M. , Fulbright, R. K. , Rajeevan, N. , Constable, R. T. , & Papademetris, X. (2008). More accurate Talairach coordinates for NeuroImaging using nonlinear registration. NeuroImage, 42, 717–725. 18572418 10.1016/j.neuroimage.2008.04.240 PMC2603575

[hbm24420-bib-0032] Lacadie, C. , Fulbright, R. K. , Constable, R. T. , & Papademetris, X. (2009). More accurate Talairach coordinates for NeuroImaging using nonlinear registration. NeuroImage, 42(2), 717–725. 10.1016/j.neuroimage.2008.04.240 PMC2603575 18572418

[hbm24420-bib-0033] Lacouture, Y. , & Cousineau, D. (2008). How to use MATLAB to fit the ex ‐ Gaussian and other probability functions to a distribution of response times. Tutorials for Quantitative Methods in Psychology, 4(1), 35–45.

[hbm24420-bib-0034] Lafon, B. , Rahman, A. , Bikson, M. , & Parra, L. C. (2017). Direct current stimulation alters neuronal input/output function. Brain Stimulation, 10(1), 36–45. 27717601 10.1016/j.brs.2016.08.014 PMC5774009

[hbm24420-bib-0035] Lefebvre, S. , & Liew, S. L. (2017). Anatomical parameters of tDCS to modulate the motor system after stroke: A review. Frontiers in Neurology, 8, 1–18. 28232816 10.3389/fneur.2017.00029 PMC5298973

[hbm24420-bib-0036] Li, L. M. , Leech, R. , Scott, G. , Malhotra, P. , Seemungal, B. , & Sharp, D. J. (2015). The effect of oppositional parietal transcranial direct current stimulation on lateralized brain functions. The European Journal of Neuroscience, 42(11), 2904–2914. 26414683 10.1111/ejn.13086 PMC4737321

[hbm24420-bib-0037] Li, L. M. , Uehara, K. , & Hanakawa, T. (2015). The contribution of interindividual factors to variability of response in transcranial direct current stimulation studies. Frontiers in Cellular Neuroscience, 9, 1–19. 26029052 10.3389/fncel.2015.00181 PMC4428123

[hbm24420-bib-0038] Logothetis, N. K. , Pauls, J. , Augath, M. , Trinath, T. , & Oeltermann, A. (2001). Neurophysiological investigation of the basis of the fMRI signal. Nature, 412, 150–157. 11449264 10.1038/35084005

[hbm24420-bib-0039] McLaren, D. G. , Ries, M. L. , Xu, G. , & Johnson, S. C. (2012). A generalized form of context‐dependent psychophysiological interactions (gPPI): A comparison to standard approaches. NeuroImage, 61(4), 1277–1286. 22484411 10.1016/j.neuroimage.2012.03.068 PMC3376181

[hbm24420-bib-0040] Meinzer, M. , Lindenberg, R. , Thy, M. , Ulm, L. , Volk, C. , & Fl, A. (2015). Transcranial direct current stimulation in mild cognitive impairment : Behavioral effects and neural mechanisms. Alzheimers & Dementia, 11, 1032–1040. 10.1016/j.jalz.2014.07.159 25449530

[hbm24420-bib-0041] Mesulam, M. (1990). Large scale neurocognitive networks and distributed processing for attention, language, and memory. Annals of Neurology, 28, 597–613. 2260847 10.1002/ana.410280502

[hbm24420-bib-0042] Monai, H. , Ohkura, M. , Tanaka, M. , Oe, Y. , Konno, A. , Hirai, H. , … Hirase, H. (2016). Calcium imaging reveals glial involvement in transcranial direct current stimulation‐induced plasticity in mouse brain. Nature Communications, 7, 11100. 10.1038/ncomms11100 PMC4804173 27000523

[hbm24420-bib-0043] Neuling, T. , Rach, S. , Herrmann, C. S. , & Schwiedrzik, C. M. (2013). Orchestrating neuronal networks : Sustained after‐effects of transcranial alternating current stimulation depend upon brain states. Frontiers in Human Neuroscience, 7(April), 1–12. 23641206 10.3389/fnhum.2013.00161 PMC3639376

[hbm24420-bib-0044] Nitsche, M. A. , & Paulus, W. (2000). Excitability changes induced in the human motor cortex by weak transcranial direct current stimulation. The Journal of Physiology, 527(3), 633–639. 10990547 10.1111/j.1469-7793.2000.t01-1-00633.x PMC2270099

[hbm24420-bib-0045] Nitsche, M. A. , Seeber, A. , Frommann, K. , Klein, C. C. , Rochford, C. , Nitsche, M. S. , … Tergau, F. (2005). Modulating parameters of excitability during and after transcranial direct current stimulation of the human motor cortex. The Journal of Physiology, 568(Pt 1), 291–303. 16002441 10.1113/jphysiol.2005.092429 PMC1474757

[hbm24420-bib-0046] Opitz, A. , Paulus, W. , Will, A. , & Thielscher, A. (2015). Determinants of the electric field during transcranial direct current stimulation. NeuroImage, 109, 2. 10.1016/j.neuroimage.2015.01.033 25613437

[hbm24420-bib-0047] O'Reilly, J. X. , Woolrich, M. W. , Behrens, T. E. J. , Smith, S. M. , & Johansen‐Berg, H. (2012). Tools of the trade: Psychophysiological interactions and functional connectivity. Social Cognitive and Affective Neuroscience, 7(5), 604–609. 22569188 10.1093/scan/nss055 PMC3375893

[hbm24420-bib-0048] Park, C. H. , Chang, W. H. , Park, J. Y. , Shin, Y. I. , Kim, S. T. , & Kim, Y. H. (2013). Transcranial direct current stimulation increases resting state interhemispheric connectivity. Neuroscience Letters, 539, 7–10. 23416318 10.1016/j.neulet.2013.01.047

[hbm24420-bib-0049] Peña‐Gómez, C. , Sala‐Lonch, R. , Junqué, C. , Clemente, I. C. , Vidal, D. , Bargalló, N. , … Bartrés‐Faz, D. (2012). Modulation of large‐scale brain networks by transcranial direct current stimulation evidenced by resting‐state functional MRI. Brain Stimulation, 5, 252–263. 21962981 10.1016/j.brs.2011.08.006 PMC3589751

[hbm24420-bib-0050] Polania, R. , Paulus, W. , & Nitsche, M. A. (2012). Reorganizing the intrinsic functional architecture of the human primary motor cortex during rest with non‐invasive cortical stimulation. PLoS One, 7(1), 1–10. 10.1371/journal.pone.0030971 PMC3267735 22303478

[hbm24420-bib-0051] Polanía, R. , Paulus, W. , & Nitsche, M. A. (2012). Modulating cortico‐striatal and thalamo‐cortical functional connectivity with transcranial direct current stimulation. Human Brain Mapping, 33(10), 2499–2508. 21922602 10.1002/hbm.21380 PMC6870027

[hbm24420-bib-0052] Purpura, D. P. , & Mcmurtry, J. G. (1965). Intracellular activities and evoked potential changes during polarizaotion of motor cortex. Journal of Neurophysiology, 28, 166–185. 14244793 10.1152/jn.1965.28.1.166

[hbm24420-bib-0053] Radman, T. , Ramos, R. L. , Brumberg, J. C. , & Bikson, M. (2009). Role of cortical cell type and morphology in sub‐ and Suprathreshold uniform electrical field stimulation. Brain Stimulation, 2(4), 215–228. 20161507 10.1016/j.brs.2009.03.007 PMC2797131

[hbm24420-bib-0054] Rahman, A. , Reato, D. , Arlotti, M. , Gasca, F. , Datta, A. , Parra, L. C. , & Bikson, M. (2013). Cellular effects of acute direct current stimulation: Somatic and synaptic terminal effects. The Journal of Physiology, 591, 2563–2578. 23478132 10.1113/jphysiol.2012.247171 PMC3678043

[hbm24420-bib-0055] Raichle, M. , Macleod, A. , Snyder, A. , Powers, W. , Gusnard, D. , & Shulman, G. (2001). A default mode of brain function. PNAS, 98, 676–682. 11209064 10.1073/pnas.98.2.676 PMC14647

[hbm24420-bib-0056] Salimi‐khorshidi, G. , Douaud, G. , Beckmann, C. F. , & Matthew, F. (2015). Automatic Denoising of functional MRI data: Combining independent component analysis and hierarchical fusion of classifiers. NeuroImage, 90(0), 449–468. 10.1016/j.neuroimage.2013.11.046 PMC4019210 24389422

[hbm24420-bib-0057] Sandrini, M. , Fertonani, A. , Cohen, L. G. , & Miniussi, C. (2012). Double dissociation of working memory load effects induced by bilateral parietal modulation. Neuropsychologia, 50(3), 396–402. 22223077 10.1016/j.neuropsychologia.2011.12.011 PMC4880015

[hbm24420-bib-0058] Sankarasubramanian, V. , Cunningham, D. , Potter, K. , Beall, E. , Roelle, S. , Varnerin, N. , … Plow, E. (2017). Transcranial direct current stimulation targeting primary motor versus dorsolateral prefrontal cortices: Proof‐of‐concept study investigating functional connectivity of thalamo‐cortical networks specific to sensory‐affective information processing. Brain Connectivity, 7(3), 182–196. 28142257 10.1089/brain.2016.0440 PMC5399740

[hbm24420-bib-0059] Sasaki, R. , Miyaguchi, S. , Kotan, S. , Kojima, S. , & Kirimoto, H. (2016). Modulation of cortical inhibitory circuits after cathodal transcranial direct current stimulation over the primary motor cortex. Frontiers in Human Neuroscience, 10, 1–8. 26869909 10.3389/fnhum.2016.00030 PMC4740366

[hbm24420-bib-0060] Seeley, W. W. , Menon, V. , Schatzberg, A. F. , Keller, J. , Glover, G. H. , Kenna, H. , … Greicius, M. D. (2007). Dissociable intrinsic connectivity networks for salience processing and executive control. The Journal of Neuroscience, 27(9), 2349–2356. 17329432 10.1523/JNEUROSCI.5587-06.2007 PMC2680293

[hbm24420-bib-0061] Sehm, B. , Kipping, J. , Schäfer, A. , Villringer, A. , & Ragert, P. (2013). A comparison between Uni‐ and bilateral tDCS effects on functional connectivity of the human motor cortex. Frontiers in Human Neuroscience, 7, 183. 23675337 10.3389/fnhum.2013.00183 PMC3646257

[hbm24420-bib-0062] Sharp, D. J. , Beckmann, C. F. , Greenwood, R. , Kinnunen, K. M. , Bonnelle, V. , De Boissezon, X. , … Leech, R. (2011). Default mode network functional and structural connectivity after traumatic brain injury. Brain, 134, 2233–2247. 21841202 10.1093/brain/awr175

[hbm24420-bib-0063] Smith, S. M. (2002). Fast robust automated brain extraction. Human Brain Mapping, 155, 143–155. 10.1002/hbm.10062 PMC6871816 12391568

[hbm24420-bib-0064] Smith, S. M. , Jenkinson, M. , Woolrich, M. W. , Beckmann, C. F. , Behrens, T. E. J. , Johansen‐berg, H. , … Matthews, P. M. (2004). Advances in functional and structural MR image analysis and implementation as FSL technical report TR04SS2. NeuroImage, 23(S1), 208–219. 10.1016/j.neuroimage.2004.07.051 15501092

[hbm24420-bib-0065] Sporns, O. (2013). Structure and function of complex brain networks. Dialogues in Clinical Neuroscience, 15(3), 247–262. 24174898 10.31887/DCNS.2013.15.3/osporns PMC3811098

[hbm24420-bib-0066] Sridharan, D. , Levitin, D. J. , & Menon, V. (2008). A critical role for the right fronto‐insular cortex in switching between central‐executive and default‐mode networks. PNAS, 105(34), 12569–12574. 18723676 10.1073/pnas.0800005105 PMC2527952

[hbm24420-bib-0067] Stagg, C. J. , Best, J. G. , Stephenson, M. C. , Shea, J. O. , Wylezinska, M. , Kincses, Z. T. , … Johansen‐berg, H. (2009). Polarity‐sensitive modulation of cortical neurotransmitters by transcranial stimulation. The Journal of Neuroscience, 29(16), 5202–5206. 19386916 10.1523/JNEUROSCI.4432-08.2009 PMC6665468

[hbm24420-bib-0068] Stagg, C. J. , & Johansen‐berg, H. (2013). Studying the effects of transcranial direct‐current stimulation in stroke recovery using magnetic resonance imaging. Frontiers in Human Neuroscience, 7, 1–8. 24376413 10.3389/fnhum.2013.00857 PMC3859898

[hbm24420-bib-0069] Stagg, C. J. , & Nitsche, M. A. (2011). Physiological basis of transcranial direct current stimulation. The Neuroscientist, 17, 37–53. 21343407 10.1177/1073858410386614

[hbm24420-bib-0070] Tazoe, T. , Endoh, T. , Kitamura, T. , & Ogata, T. (2014). Polarity specific effects of transcranial direct current stimulation on interhemispheric inhibition. PLoS One, 9, e114244. 25478912 10.1371/journal.pone.0114244 PMC4257682

[hbm24420-bib-0071] Thielscher, A. , Antunes, A. , & Saturnino, G. B. (2015). Field modeling for transcranial magnetic stimulation: A useful tool to understand the physiological effects of TMS? IEEE Transactions on Biomedical Engineering, 2015, 222–225. 10.1109/EMBC.2015.7318340 26736240

[hbm24420-bib-0072] Turski, C. , Kessler‐Jones, A. , Chow, C. , Hermann, B. , Hsu, D. , Jones, J. , … Ikonomidou, C. (2017). Extended multiple‐field high‐definition transcranial direct current stimulation (HD‐TDCS) is well tolerated and safe in healthy adults. Restorative Neurology and Neuroscience, 35(6), 631–642. 29172010 10.3233/RNN-170757 PMC5730273

[hbm24420-bib-0073] Vandermeeren, Y. , Jamart, J. , & Ossemann, M. (2010). Effect of tDCS with an extracephalic reference electrode on cardio‐respiratory and autonomic functions. BMC Neuroscience, 11(38), 1–10. 10.1186/1471-2202-11-38 PMC2844382 20233439

[hbm24420-bib-0074] Vatansever, D. , Manktelow, A. E. , Sahakian, B. J. , Menon, D. K. , & Stamatakis, E. A. (2017). Angular default mode network connectivity across working memory load. Human Brain Mapping, 52, 41–52. 10.1002/hbm.23341 PMC6866899 27489137

[hbm24420-bib-0075] Vatansever, D. , Menon, D. K. , Manktelow, A. E. , Sahakian, B. J. , & Stamatakis, E. A. (2015). Default mode network connectivity during task execution. NeuroImage, 122, 96–104. 26220743 10.1016/j.neuroimage.2015.07.053

[hbm24420-bib-0076] Violante, I. R. , Li, L. M. , Carmichael, D. W. , Lorenz, R. , Leech, R. , Hampshire, A. , … Sharp, D. J. (2017). Externally induced frontoparietal synchronization modulates network dynamics and enhances working memory performance. eLife, 6, e22001. 28288700 10.7554/eLife.22001 PMC5349849

[hbm24420-bib-0077] Vosskuhl, J. , Huster, R. J. , & Herrmann, C. S. (2015). BOLD signal effects of transcranial alternating current stimulation (tACS) in the alpha range: A concurrent tACS‐fMRI study. NeuroImage, 140, 118–125. 26458516 10.1016/j.neuroimage.2015.10.003

[hbm24420-bib-0078] Wiethoff, S. , Hamada, M. , & Rothwell, J. C. (2014). Variability in response to transcranial direct current stimulation of the motor cortex. Brain Stimulation, 7(3), 468–475. 24630848 10.1016/j.brs.2014.02.003

[hbm24420-bib-0079] Windhoff, M. , Opitz, A. , & Thielscher, A. (2013). Electric field calculations in brain stimulation based on finite elements: An optimized processing pipeline for the generation and usage of accurate individual head models. Human Brain Mapping, 34(4), 923–935. 22109746 10.1002/hbm.21479 PMC6870291

[hbm24420-bib-0080] Wu, Y.‐J. , Tseng, P. , Chang, C.‐F. , Pai, M.‐C. , Hsu, K.‐S. , Lin, C.‐C. , & Juan, C.‐H. (2014). Modulating the interference effect on spatial working memory by applying transcranial direct current stimulation over the right dorsolateral prefrontal cortex. Brain Cognition, 91, 87–94. 25265321 10.1016/j.bandc.2014.09.002

